# Place of birth and postnatal transfers in infants with congenital diaphragmatic hernia in England and Wales: a descriptive observational cohort study

**DOI:** 10.1136/archdischild-2023-326152

**Published:** 2024-02-05

**Authors:** Behrouz Nezafat Maldonado, Julia Lanoue, Benjamin Allin, Dougal Hargreaves, Marian Knight, Chris Gale, Cheryl Battersby

**Affiliations:** 1 Neonatal Medicine, Faculty of Medicine, School of Public Health, Imperial College London, Chelsea and Westminster Campus, London, UK; 2 Centre for Paediatrics and Child Health, Imperial College London, London, UK; 3 National Perinatal Epidemiology Unit, University of Oxford, Oxford, UK; 4 Department of Primary Care and Public Health, Imperial College London, London, UK

**Keywords:** Neonatology, Child Health Services

## Abstract

**Objective:**

To describe clinical pathways for infants with congenital diaphragmatic hernia (CDH) and short-term outcomes.

**Design:**

Retrospective observational cohort study using the UK National Neonatal Research Database (NNRD).

**Patients:**

Babies with a diagnosis of CDH admitted to a neonatal unit in England and Wales between 2012 and 2020.

**Main outcome measures:**

Clinical pathways defined by place of birth (with or without colocated neonatal and surgical facilities), transfers, clinical interventions, length of hospital stay and discharge outcome.

**Results:**

There were 1319 babies with a diagnosis of CDH cared for in four clinical pathways: born in maternity units with (1) colocated tertiary neonatal and surgical units (‘*neonatal surgical units*’), 50% (660/1319); (2) designated tertiary neonatal unit and transfer to stand-alone surgical centre (‘*tertiary designated*’), 25% (337/1319); (3) non-designated tertiary neonatal unit (‘*tertiary non-designated’*), 7% (89/1319); or (4) non-tertiary unit (‘*non-tertiary*’), 18% (233/1319)—the latter three needing postnatal transfers. Infant characteristics were similar for infants born in *neonatal surgical* and *tertiary designated* units. Excluding 149 infants with minimal data due to early transfer (median (IQR) 2.2 (0.4–4.5) days) to other settings, survival to neonatal discharge was 73% (851/1170), with a median (IQR) stay of 26 (16–44) days.

**Conclusions:**

We found that half of the babies with CDH were born in hospitals that did not have on-site surgical services and required postnatal transfer. Similar characteristics between infants born in neonatal surgical units and tertiary designated units suggest that organisation rather than infant factors influence place of birth. Future work linking the NNRD to other datasets will enable comparisons between care pathways.

**WHAT IS ALREADY KNOWN ON THIS TOPIC**

- National guidelines recommend that infants with known surgical anomalies are delivered in maternity units with colocated neonatal medical and surgical units.

**WHAT THIS STUDY ADDS**

- Each year, around 147 babies with congenital diaphragmatic hernia (CDH) are admitted to neonatal units in England and Wales. Half of them are born in maternity units without an on-site surgical facility and consequently require postnatal transfer.
- Similar characteristics between those born in neonatal surgical units and tertiary designated units suggest that organisation rather than infant factors influence place of birth.
- Out of ten babies, around seven will survive neonatal discharge, half are discharged home and a quarter are discharged to other paediatric settings for ongoing care.

**HOW THIS STUDY MIGHT AFFECT RESEARCH, PRACTICE OR POLICY**

- We identified four different clinical pathways in England and Wales that may lead to variation in care and outcomes of babies with CDH.
- Data linkage between available health datasets is urgently needed to reliably evaluate factors that influence outcomes including the organisation of services.
- This would facilitate future research needed to determine whether variation in place of birth and care pathways observed in this study influence short and long-term outcomes of babies with CDH.

## Background

Congenital diaphragmatic hernia (CDH), a defect in the formation of the diaphragm, can result in abdominal organs herniating into the chest compromising lung development.^1 2^ This defect may manifest as poor lung function at birth, respiratory failure and death. Advancements in prenatal diagnosis, neonatal interventions and surgical techniques have improved outcomes of infants with CDH.^3 4^

Antenatal screening aims to detect CDH early.^5^ In the UK, around 60% of CDH cases are antenatally diagnosed.^6^ This allows for parental counselling and shared decision-making on place of birth. However, evidence gaps on the optimal timing and place of birth can hinder informed decision-making.^7^ It is necessary to evaluate how neonatal services are organised to ensure infants with CDH are cared for in appropriately resourced settings.^7^

National guidelines recommend that neonates requiring surgical care are born in maternity centres with colocated neonatal surgery.^8 9^ UK neonatal services are organised in networks.^10^ In several networks, neonatal surgery is provided in ‘stand-alone surgical units’ without colocated maternity. Babies born in these networks are transferred postnatally. Previous research in England found that lack of colocation leads to avoidable postnatal transfers, with estimates of 31 cases of CDH undergoing an avoidable postnatal transfer annually.^7 11^

There is mixed evidence on whether place of birth for CDH impacts outcomes.^12^ The role of place of birth in short-term outcomes has not been studied previously in the UK. Examining this relationship is complex, as both place of birth and outcomes are associated with an array of demographic, clinical and organisational factors. However, routine data can potentially be used to examine the relationship between these factors and infant outcomes at a population level.

The National Neonatal Research Database (NNRD) comprises quality-assured data on all infants admitted to UK neonatal units.^13^ The NNRD enables us to study admissions of infants from birth to discharge from neonatal care. However, the NNRD does not capture care in other settings such as stand-alone paediatric surgical centres, paediatric intensive care or paediatric wards.

We aimed to describe current care pathways defined by place of birth for infants with CDH born in England and Wales and describe interventions and short-term outcomes.

## Methods

### Study design, setting and participants

We conducted the study in a two-stage process.

Stage 1: Discussions with experts in neonatal medicine, paediatric intensive care and neonatal surgery to explore care pathways for infants with CDH.

Stage 2: A retrospective observational study using routinely recorded data from the NNRD. Data were used to explore care pathways and short-term health outcomes. We report in line with Reporting of Studies Conducted Using Observational Routinely Collected Health Data guidelines.^14^

### Population

Data were extracted for all infants admitted to a neonatal unit in England and Wales between 1 January 2012 and 31 December 2020 with a diagnosis of CDH (as defined in online supplemental appendix). We excluded infants with inconsistent transfer patterns and those diagnosed with multiple congenital (non-cardiac) surgical abnormalities, for example, CDH and gastroschisis.

10.1136/fetalneonatal-2023-326152.supp1 Supplementary data

We used Office for National Statistics data on live births in England and Wales across the study period to estimate the prevalence of CDH.^15^

### Outcomes

We report primary outcome survival to discharge to home and neonatal discharge to other settings. Secondary outcome measures include postnatal management, transfer patterns and length of stay (table 1).

**Table 1:** Outcome measures of interest, definition and how they were derived

Outcome measure	Description	Source	Question	Rationale
Care pathways	Colocated maternity unit (place of birth) with neonatal and surgical units, or postnatal transfer via ambulance to surgical centre	Semistructured interviews, focus groups and routine data analysis	Where are infants with CDH delivered and are intensive care and surgical services present at the delivery unit?	Identify care pathways.
Received antenatal care	Number of pregnancies that were booked	Routine data on admission	Was the pregnancy booked?	If no antenatal care, likely defect was postnatally diagnosed.
Admission <2 hours from birth	Proportion of infants admitted to a neonatal unit within 2 hours from birth. This acts as a proxy measure for antenatal diagnosis.	Routine data on admission	What proportion of infants are admitted to neonatal unit within 2 hours from birth?	Proxy for antenatal diagnosis
Median age of admission	Minutes from birth to neonatal unit admission	Routine data on admission	When did the admission occur?	Proxy for antenatal diagnosis
Drugs at delivery	Proportion of infants receiving drugs at resus including epinephrine	Routine data on admission	Were resuscitation drugs needed at delivery?	Proxy for disease severity
Inotropes on day 1	Number of infants receiving inotropes on day 1	Routine data on daily care	Were inotropes used on day 1?	
Inhaled nitric oxide on day 1	Number of infants receiving inhaled nitric oxide on day 1	Routine data on daily care	Was inhaled nitric oxide used on day 1?	
Mechanical ventilation on day 1	Number of infants on mechanical ventilation on day 1	Routine data on daily care	Was mechanical ventilation used on day 1?	
Transfer patterns	Proportion of postnatal transfers that occurred at 24, 48 and 72 hours from birth	Routine data on discharge	What proportion of infants are transferred out of the first neonatal unit at 24, 48 and 72 hours?	Identify what transfers occur due to the place of birth.
Time of transfer to surgical unit	For those infants born in a not colocated centre we report the median age at transfer.	Routine data on discharge		
Ventilation mode during neonatal stay	Ventilation mode received—conventional, high-frequency oscillation or multiple modes. Proxy measure for disease severity.	Routine data on daily care	What ventilation modes are used for CDH management?	Proxy for disease severity
Prostaglandin use during neonatal unit stay	Use of prostaglandin during stay in neonatal unit	Routine data on daily care	Was prostaglandin given during neonatal stay?	
Inhaled nitric oxide use during neonatal unit stay	Use of inhaled nitric oxide during stay in neonatal unit	Routine data on daily care	Was inhaled nitric oxide given during neonatal stay?	
Sildenafil use during neonatal unit stay	Use of sildenafil during stay in neonatal unit	Routine data on daily care	Was sildenafil given during neonatal stay?	
Surfactant use during neonatal unit stay	Use of prostaglandin during stay in neonatal unit	Routine data on daily care	Was surfactant given during neonatal stay?	
Extracorporeal membrane oxygenation (ECMO) use	Use of ECMO or discharge for ECMO during stay in neonatal unit	Routine data on daily care and discharge details	Did the infant have ECMO or were they discharged from a neonatal unit for ECMO?	
Length of neonatal stay	Time from birth to discharge from neonatal unit	Routine data on discharge details		Describe outcomes across care pathways.
Discharge to other settings from neonatal unit	Destination after neonatal episode recorded in NNRD ended: paediatric ward specialist care, for example, cardiac centre, surgical centre or paediatric intensive care. Local repatriation.		Where are infants discharged to?	
Survival to surgical centre	Survived and transferred to surgical centre			
Survival to discharge from neonatal unit	Survived neonatal stay			

CDH, congenital diaphragmatic hernia; NNRD, National Neonatal Research Database.

We define postnatal transfer as any transfer that requires ambulance transport. We sought clarification from the expert advisory panel on which units required postnatal transfer between neonatal and surgical units and sought consensus. Transfers were identified from the NNRD discharge destination code.

### Statistical analysis

We report findings across clinical pathways and present descriptive statistics using median/IQR and percentage as appropriate. We report survival to neonatal discharge, discharge destination and length of hospital stay. This is a descriptive study and we have not undertaken any formal analysis to compare outcomes between pathways or adjust confounders. All analyses were performed using R V.3.6.

### Research ethics and other approvals

We used deidentified data from the NNRD.^13 16^ All neonatal units agreed to the inclusion of their data in the study.

## Results

### Stage 1: establish expert advisory board

Experts from across 6 out of 10 neonatal networks in England and Wales participated in the expert advisory board. Members included six neonatologists, eight neonatal surgeons and one paediatric cardiac intensivist. They worked in either tertiary neonatal units with colocated surgery or tertiary neonatal units without surgery on-site. The four pathways identified were based on birth in a maternity unit with:

1. Colocated tertiary neonatal and surgical units (*neonatal surgical units*).
2. Designated tertiary neonatal unit for surgical conditions and transfer to a surgical centre (*tertiary designated*).
3. Tertiary neonatal unit not designated for surgical conditions and transfer to a surgical centre (*tertiary non-designated*).
4. Non-tertiary units without surgery and transfer to a surgical centre (*non-tertiary*).

Birth in *neonatal surgical units* (1) is the only pathway that does not require postnatal transfer in an ambulance to a surgical unit.

### Stage 2: routine data analysis using NNRD

**Figure 1:**
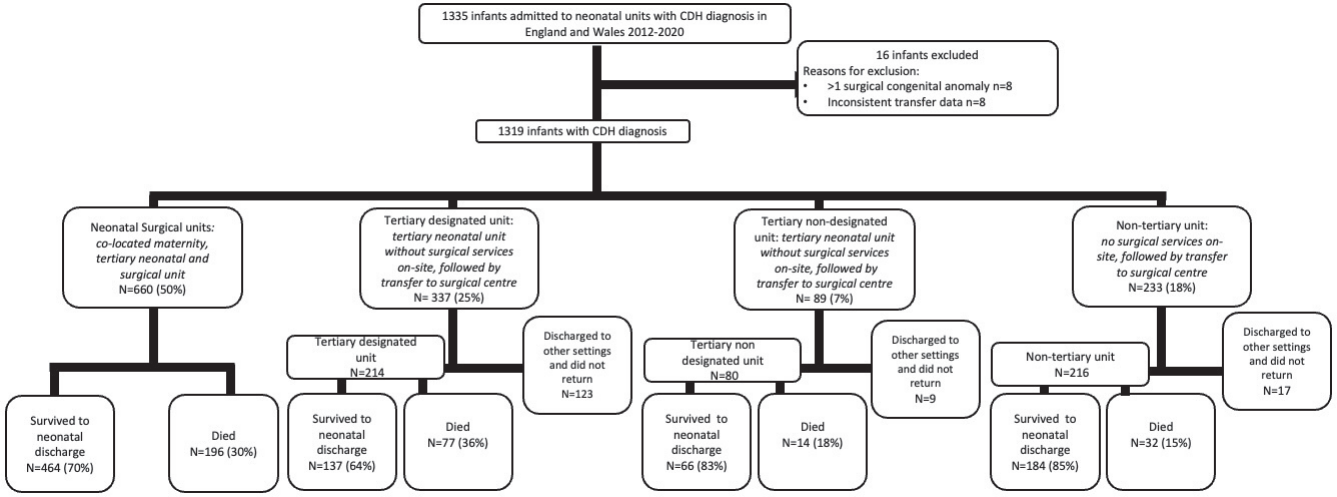
Study population, clinical pathways defined by place of birth and *survival to neonatal discharge*. Number and proportion of infants admitted to each type of neonatal unit at birth displayed, together with the outcome of their neonatal stay: survival to neonatal discharge, death or discharge to other settings early not returning to an NNRD unit. CDH, congenital diaphragmatic hernia.

Between 1 January 2012 and 31 December 2020, a total of 1335 babies with a diagnosis of CDH were admitted to a neonatal unit in England and Wales of which 16 were excluded from the study (figure 1). There were 6 108 030 live births during this period in England and Wales, resulting in an estimated live birth incidence of 2.2 per 10 000 (95% CI 2.1 to 2.3). The true live birth incidence is likely to be higher as our case number excludes babies born alive who died in the delivery room.

### Clinical pathways

**Figure 2:**
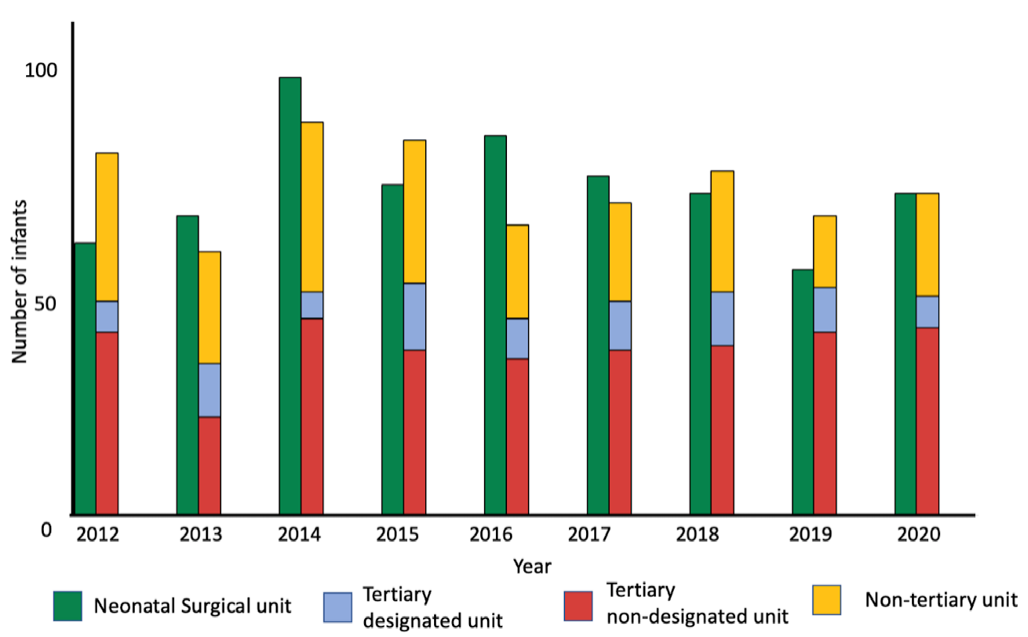
Number of cases per year born in maternity units with neonatal surgical units (green) and non-colocated units (stacked bar): designated tertiary units (blue), non-designated tertiary intensive care units (red) and non-tertiary units (yellow).

We identified 16 *neonatal surgical units*, 5 *tertiary designated* units and 5 stand-alone surgical centres without an on-site neonatal unit across England and Wales. During the study period 660/1319 (50%) infants were delivered in *neonatal surgical units*. The other half required postnatal transfer to a surgical unit (figure 1). We report yearly births across each clinical pathway during the study period (figure 2). We found that a median of 72 (67–76) of 146 cases were born in *neonatal surgical units* annually.

### Infant and maternal characteristics

Table 2 summarises infant and maternal characteristics by pathway. Infants were most commonly male with a median gestational age of 38 weeks. Across the cohort, median (IQR) maternal age was 30.5 (26–35) years and half of the group were white British; between 10% and 20% of maternal ethnicity data were missing.

**Table 2:** Baseline characteristics and first transfer details by place of birth

Place of birth	Neonatal surgical unit, n=660	Tertiary designated unit, n=337	Tertiary non-designated unit, n=89	Non-tertiary unit, n=233
**Infant characteristics at birth**				
<28 weeks’ gestation, n (%)	5 (1)	2 (1)	2 (2)	1 (<1)
≥28 to 32 weeks’ gestation, n (%)	30 (4)	5 (1)	5 (6)	20 (9)
≥32 to 36+6 weeks’ gestation, n (%)	132 (20)	63 (19)	20 (22)	46 (20)
>37 weeks’ gestation, n (%)	493 (75)	267 (79)	62 (70)	166 (71)
Gestation in weeks, median (IQR)	38 (36, 39)	38 (37, 39)	38 (35, 40)	38 (36, 40)
Girls, n (%)	279 (42)	136 (40)	38 (43)	83 (36)
Multiple birth, n (%)	34 (5)	16 (5)	5 (6)	8 (4)
Birth weight (g), median (IQR)	2897 (2444, 3250)	3000 (2575, 3370)	2960 (2280, 3442)	3080 (2420, 3470)
Caesarean delivery, n (%)	241 (38)	120 (36)	36 (40)	82 (35)
*Missing*, n (%)	46 (7)	9 (3)	5 (6)	3 (1)
**Maternal characteristics**				
Maternal age, median (IQR)	31.0 (27.0, 35.0)	30.0 (26.0, 34.0)	30.0 (26.0, 34.0)	31.0 (27.0, 35.0)
*Missing*, n (%)	4 (<1)	4 (2)	2 (2)	0 (0)
Index of Multiple Deprivation decile, median (IQR)	5.00 (2.00, 7.00)	3.50 (2.00, 6.00)	3.00 (2.00, 6.00)	5.00 (3.00, 8.00)
*Missing*, n (%)	12 (2)	7 (2)	0 (0)	4 (2)
Maternal ethnicity				
White British, n (%)	337 (51)	183 (54)	52 (58)	116 (50)
*Missing*, n (%)	95 (14)	71 (21)	10 (11)	46 (20)
Maternal gestational diabetes, n (%)	41 (6)	21 (6)	4 (5)	15 (6)
Smoking in pregnancy, n (%)	78 (12)	46 (14)	15 (17)	30 (13)
*Missing*, n (%)	97 (15)	35 (10)	8 (9)	25 (11)
Received antenatal care, n (%)	486 (74)	225 (67)	68 (76)	162 (68)
*Missing*, n (%)	113 (17)	78 (20)	12 (13)	58 (24)
Apgar score >7 at 5 min, n (%)	358 (54)	185 (55)	50 (56)	130 (55)
**Organisational factors**				
Admission to neonatal unit <2 hours from birth, n (%)	627 (95)	330 (98)	69 (78)	175 (74)
Median time admission from birth, min (IQR)	25 (17–35)	18 (14–25)	34 (24–82)	43 (25–125)
Transfer to another hospital at <24 hours from birth, n (%)	16 (2)	32 (10)	53 (60)	191 (81)
Transfer to another hospital at 24–48 hours from birth, n (%)	22 (3)	40 (12)	9 (10)	13 (6)
Transfer to another hospital at 48–72 hours from birth, n (%)	3 (0.5)	41 (12)	6 (7)	6 (3)
Median age transfer to surgical unit, days	N/A	4 (1.8–4)	1 (0.3–1.6)	0.5 (0.3–0.6)
Transferred to a children’s hospital—including stand-alone surgical units, n (%)	61 (9)	241 (72)	27 (30)	67 (29)
Received all care in neonatal unit	499 (76)	121 (36)	52 (58)	163 (70)
**Intervention on day 1**				
Received drugs at birth, n (%)	133 (20)	34 (10)	4 (4.5)	2 (0.8)
Received inotropes on day 1, n (%)	332 (50)	169 (50)	27 (30)	61 (26)
Received nitric oxide on day 1, n (%)	287 (43)	149 (44)	22 (25)	25 (11)
Received mechanical ventilation on day 1, n (%)	601 (91)	309 (92)	66 (74)	148 (64)
**ECMO use during neonatal stay**				
Received ECMO or discharge from unit for ECMO, n (%)	54 (8)	28 (8)	8 (9)	8 (3)
Median age ECMO transfer, days (IQR)	1.3 (0.9–2.1)	1.1 (0.6–1.8)	1 (0.5–4.3)	1.5 (0.8–1.8)
**Ventilation mode during NNU stay**				
Conventional ventilation only, n (%)	216 (45)	49 (24)*	53 (70)*	160 (80)*
High-frequency oscillation only, n (%)	73 (15)	39 (19)*	2 (2.6)*	4 (2.0)*
Multiple modes of ventilation, n (%)	195 (40)	114 (53)*	21 (28)*	37 (18)*
*Unknown*, n (%)	15 (3)	12 (5)*	4 (5)	15 (7)*
**Surfactant administration outside of delivery room**				
Surfactant given, yes, n (%)	70 (14)	38 (18)*	25 (31)*	54 (25)*
**Sildenafil use during NNU stay**				
Sildenafil given, yes, n (%)	24 (5)	14 (6.5)*	4 (5.0)*	10 (4.6)*
**Inhaled nitric oxide during NNU stay**				
Inhaled nitric oxide given, yes, n (%)	283 (57)	140 (66)*	33 (41)*	67 (31)*
**Prostaglandin use during NNU stay**				
Prostaglandin given, yes, n (%)	41 (8)	44 (21)*	8 (10)*	14 (6.5)*

*Reported for 1170 infants. Tertiary designated n=214, tertiary non-designated n=80, non-tertiary unit n=216.

ECMO, extracorporeal membrane oxygenation; NNU, neonatal unit.

### Organisational factors and postnatal transfers

More than 95% of infants born in *surgical neonatal* or *tertiary designated* units were admitted to the neonatal unit within the first 2 hours after birth compared with 74–78% in *tertiary non-designated* or *non-tertiary* units. Transfer to a surgical centre occurred within the first 24 hours in 81% in *non-tertiary centre* and 60% in a *tertiary non-designated centre*. Infants in *tertiary designated* units were transferred later, commonly after day 3 from birth (median 4 (1.8–8) days). Infants in *tertiary designated units* were transferred to a stand-alone surgical unit without a neonatal unit in 71% (239/337) cases. One *tertiary designated* centre transferred infants to the surgical unit for surgery and then retrieved them after surgery. Infants born at this unit were not discharged from their electronic health system despite being transferred to a surgical unit as preoperative care and postoperative care occur within the tertiary designated neonatal unit. Across the cohort, 30% of infants (396/1319) were postnatally transferred to a stand-alone surgical unit.

### Day 1 postnatal management

Infants in *surgical* and *tertiary designated* units received intensive care support at similar rates on day 1 including invasive ventilation (601/660, 91%; 309/337, 92%), inotropes (332/660, 50%; 169/337, 50%) and nitric oxide (287/660, 43%; 149/337, 44%). These interventions were received at lower rates in the *tertiary non-designated* and *non-tertiary* groups. Of those infants in *tertiary non-designated* units, 74% (66/89) received mechanical ventilation on day 1, 30% (27/89) received inotropes and 25% (22/89) received nitric oxide.

### Survival to a surgical centre

We found 659 infants born outside a surgical centre, of which 86% (565/659) survived to transfer to be admitted to a surgical centre. Survival to discharge to the surgical centre was higher in the *tertiary non-designated* (82/89 (92%)) and *non-tertiary unit* groups (222/233 (95%)) (online supplemental appendix).

### Discharge destination

**Figure 3:**
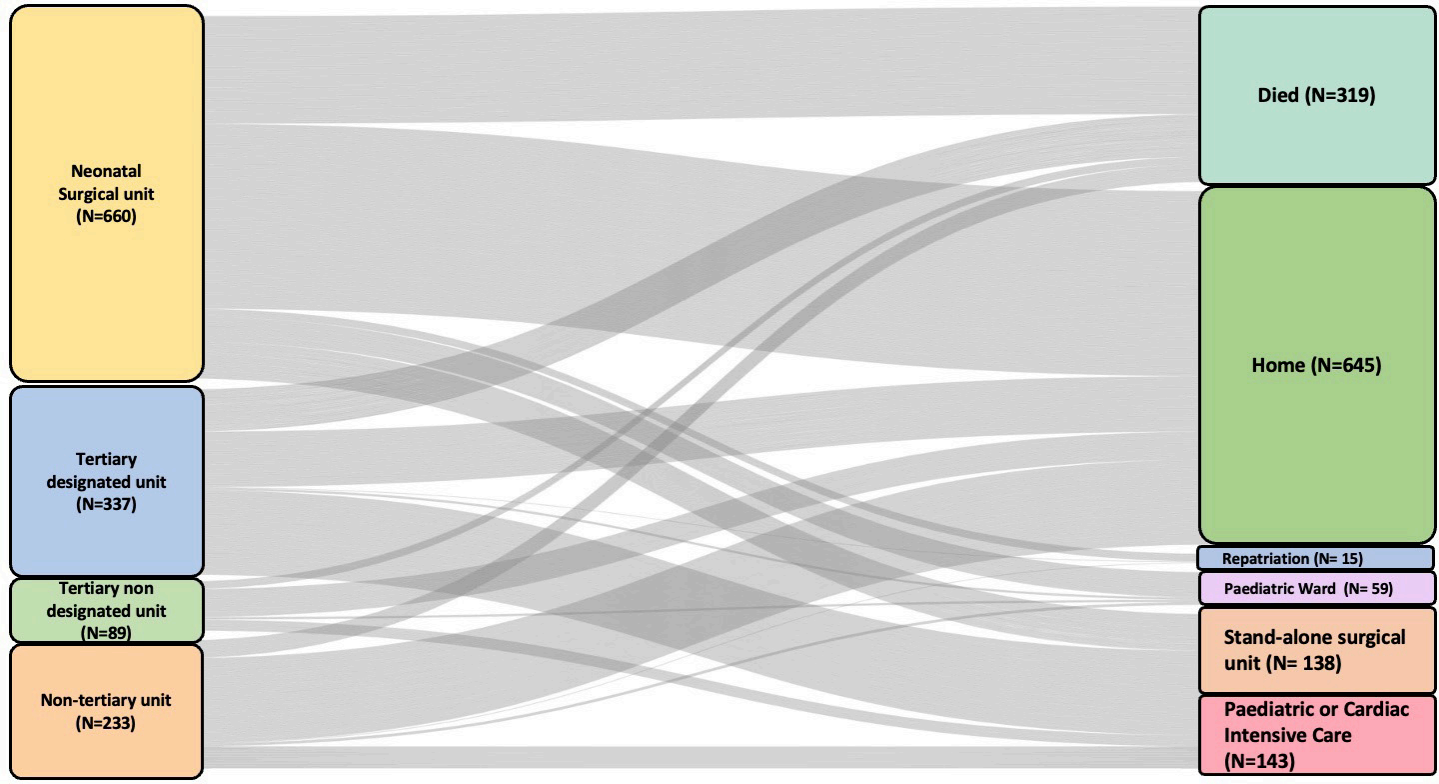
Sankey diagram with the final discharge destination recorded in the dataset for all infants across the four pathways (n=1319).

We report final neonatal discharge destination for all infants (figure 3). Among the whole cohort, 76% (1000/1319) survived neonatal discharge. 49% (645/1319) were discharged home and 27% (355/1319) were discharged to other settings. Of those discharged to other settings, 40% (143/355) were discharged to a paediatric or cardiac intensive care unit, 39% (138/355) to a stand-alone surgical unit, 17% (59/355) to a paediatric ward and 4% (15/355) to their local hospital (table 3).

**Table 3:** Discharge destination, length of stay and survival to neonatal discharge for whole population (n=1319)

	Place of birth			
	Neonatal surgical unit, n=660	Tertiary designated unit, n=337	Tertiary non-designated unit, n=89	Non-tertiary unit, n=233
Survived neonatal discharge, n (%)	464 (70)	260 (77)	75 (84)	201 (86)
Discharge destination from neonatal care, n (%)				
Paediatric ward	46 (7)	4 (1)	4 (5)	5 (2)
Stand-alone surgical centre	16 (2)	87 (26)	12 (13)	23 (10)
PICU/CICU	51 (8)	67 (20)	8 (9)	17 (7)
Repatriation local hospital	13 (2)	1 (<1)	0	1 (<1)
Home	338 (51)	101 (30)	51 (57)	155 (67)
Length of neonatal stay (survival group), median days (IQR)	27 (16–47)	14 (2–34)	19 (12–30)	19 (11–32)

CICU, cardiac intensive care unit; PICU, paediatric intensive care unit.

### Survival to neonatal discharge and postnatal management during neonatal stay

In the first days after birth, 149 infants were transferred to a stand-alone surgical centre or other paediatric settings and did not return to an NNRD contributing unit. Transfers occurred on a median of day 2 (median (IQR) 2.2. (0.9–4.5) days) (online supplemental appendix) and these infants had minimal data. We therefore present in additional data reporting survival to neonatal discharge and postnatal management for 1170 infants (89% of the original cohort) excluding these 149 infants.

We present postnatal management for 1170 infants that received the majority of care in a neonatal unit (table 2). Across all groups, infants commonly received a combination of multiple modes of ventilation during their neonatal stay. We found higher rates of inhaled nitric oxide and sildenafil use in *neonatal surgical* units and *tertiary designated* units. Surfactant was given outside the delivery room to a similar proportion of infants across all groups.

Of the 1170 infants, 73% (851/1170) survived neonatal discharge, 55% (645/1170) were discharged home and 18% (206/1170) were discharged to other settings. Of those discharged to other settings, 43% (88/206) were discharged to a paediatric or cardiac intensive care unit, 21% (44/206) went to a stand-alone surgical unit, 29% (59/206) were discharged to a paediatric ward and 7% (15/206) were discharged to their local hospital (table 4). Across this cohort, the median (IQR) hospitalisation was 25.5 (16–43.6) days.

**Table 4:** Discharge destination, length of stay and survival to neonatal discharge excluding infants with minimal data transferred early to stand-alone units (n=1170)

	Place of birth			
	Colocated neonatal unit, n=660	Tertiary designated unit, n=214*	Tertiary non-designated unit, n=80*	Non-tertiary unit, n=216*
Survived neonatal discharge, n (%)	464 (70)	137 (64)	66 (83)	184 (85)
Discharge destination from neonatal care, n (%)				
Paediatric ward	46 (7)	4 (2)	4 (5)	5 (2)
Stand-alone surgical centre	16 (2)	14 (7)	5 (6)	9 (4)
PICU/CICU	51 (8)	17 (8)	6 (8)	14 (6)
Repatriation local hospital	13 (2)	1 (<1)	0	1 (<1)
Home	338 (51)	101 (47)	51 (64)	155 (72)
Length of neonatal stay (survival group), median days (IQR)	27 (16–47)	32 (20–45)	20 (16–35)	20 (13–34)

*Total population n=1170 (89% of whole cohort).

CICU, cardiac intensive care unit; PICU, paediatric intensive care unit.

## Discussion

Over a 9-year period, 1319 infants with CDH were admitted to neonatal units in England and Wales. We identified four clinical pathways of care for neonates with a diagnosis of CDH. Half of the babies were born in maternity units with colocated neonatal surgical units and a quarter in *tertiary designated units* requiring postnatal transfer to stand-alone surgical centres. The transfer from *tertiary designated centres* to a surgical centre occurred at a median age of 4 days. The remaining quarter were born outside of these designated pathways.

Infant characteristics and rates of intensive care support were similar for infants born in *neonatal surgical* units and in *tertiary designated* units. This suggests that organisational rather than infant factors influence place of birth and care pathway, particularly for babies with antenatally diagnosed CDH, who would be predominantly cared for across these two designated pathways. We report a survival rate of 73%, consistent with previous data from England which estimated 1-year survival between 68% and 81%.^17 18^

While comparison of survival outcomes between pathways is of interest, this was not undertaken formally in this descriptive study due to the unavailability of important confounders and mediators. These include information on fetoscopic endoluminal tracheal occlusion, a procedure which has been shown to improve survival to discharge in infants with severe left-sided CDH.^19^ Importantly, we lack information on whether CDH was antenatally or postnatally diagnosed, the laterality of the defect, defect type, lung-head ratio, antenatal treatment, timing of surgery and surgical complications. We speculate, for example, that the population of babies born in the *tertiary non-designated* and *non-tertiary* groups are likely to have been postnatally diagnosed due to smaller defects being undiagnosed antenatally and hence born outside a surgical centre. This would explain the more favourable survival to neonatal discharge and the shorter length of stay in the *tertiary non-designated* and *non-tertiary* groups.

Previous data from the USA have identified that being ‘inborn’ at the treatment centre is associated with mortality in CDH,^20^ this is consistent with our findings. Whether there is a difference seen between the survival to neonatal discharge and length of stay between *neonatal surgical* unit and the *tertiary designated* unit groups warrants further exploration but requires data linkage in the UK to obtain additional information to enable case-mix adjustment.

Limitations to the study include missing data beyond the first few days of life for over one-third of babies born in tertiary designated centres transferred early to stand-alone surgical centres. To assess the impact of the missing data, we additionally reported outcomes for a subgroup of 1170 babies, excluding 149 babies (11% of the cohort), length of neonatal stay becomes longer and more consistent across the pathways in the subgroup. However, survival rate to neonatal discharge decreased for infants in *tertiary designated* units from 75% (260/337) to 64% (137/214). We speculate this is due to the disproportionate representation of deaths due to the inclusion of early mortality before transfer to a surgical centre, but exclusion of survivors transferred early to a surgical centre. The population in the subgroup may represent more severe CDH, particularly in the *tertiary designated unit* group.

A further limitation of the NNRD is that it captures data on neonatal unit admissions only and therefore while we found an estimated live birth prevalence of 2.2 per 10 000 (95% CI 2.1 to 2.3), this does not consider terminations of pregnancy or delivery room deaths.^17 21^

Strengths of our study include the population-level coverage, including all babies with CDH admitted to neonatal units in England and Wales across a 9-year period. In England and Wales, babies with CDH will be admitted to a neonatal unit following birth as their first hospital episode, unless the antenatal plan is for palliative care on the postnatal ward, or the infant does not survive birth or the CDH is not detected prior to postnatal discharge. All other infants, even if they are transferred to a non-neonatal unit for ongoing care, are included thus reducing recruitment bias.

We have demonstrated the feasibility of using routinely collected data to identify the cohort of infants with CDH receiving care in the UK. Future research aimed at informing the configuration of care pathways and determining the optimal place of birth for babies with CDH must include outcome data from stand-alone surgical centres, as well as report on long-term health and education outcomes. To improve the accuracy and completeness of data and allow for more robust conclusions to be drawn, there are plans to link data from the NNRD with other sources of routine health data, such as the National Congenital Anomaly and Rare Disease Registration Service, Hospital Episodes Statistics and educational outcomes from the National Pupil Database for this population.^22^ This data linkage will enable future studies to explore the impact of birth location on outcomes of CDH while considering all confounders. To further enhance data quality, we recommend that centres carrying out neonatal surgery, including stand-alone centres, contribute to surgical datasets or registries to enable national audits and service evaluation.

## Data Availability

Data may be obtained from a third party and are not publicly available.
