# Supplementary material for: Place of birth and postnatal transfers in infants with congenital diaphragmatic hernia in England and Wales: a descriptive observational cohort study
Source: Arch Dis Child Fetal Neonatal Ed. 2024 Feb 5;109(5):542–9. doi: 10.1136/archdischild-2023-326152 (PMC11347235; doi:10.1136/archdischild-2023-326152)
Supplement: Supplementary data [file fetalneonatal-2023-326152supp001.pdf]

Supplementary Appendix

Contents

Supplement 1: Expert group and explorative work	Page 2
Supplement 2: Diagnostic codes for CDH	Page 3
Supplement 3: Discharge destination, number of neonatal episodes and length of neonatal stay for infants born outside a neonatal surgical unit	Page 4
Supplement 4: Survival to surgical centre for the whole cohort	Page 5
Supplement 5: Sub-analysis of discharge outcomes	Page 6
Supplement 6: UK Neonatal Collaborative units in England and Wales	Page 7

Nezafat Maldonado B, *et al. Arch Dis Child Fetal Neonatal Ed* 2024; 109:542–549. doi: 10.1136/archdischild-2023-326152

Supplement 1: Expert group and explorative work

We engaged healthcare professionals including neonatologists and neonatal surgeons from ten UK operational delivery networks. We did not involve patients or members of the public due to the remit of this work being to study the current health system pathways.

Meetings took place virtually and included both 1-1 interviews and group interviews, all facilitated by BNM. During the meeting, we discussed the different pathways for babies with congenital anomalies across England and Wales.

**Role**	**Neonatal Network**
Consultant Paediatric and Neonatal Surgeon	Thames Valley & Wessex
Clinical Research Fellow in Paediatric Surgery	Thames Valley & Wessex
Consultant Paediatric and Neonatal Surgeon	North West
Consultant Paediatric and Neonatal Surgeon	North West
Clinical Research Fellow in Paediatric Surgery	North West
Consultant Paediatric and Neonatal Surgeon	London
Consultant Paediatric and Neonatal Surgeon	West Midlands
Consultant Paediatric and Neonatal Surgeon	London
Consultant Paediatric Intensive Care	London
Consultant Neonatologist	South West
Consultant Neonatologist	London
Consultant Neonatologist	Thames Valley & Wessex
Consultant Neonatologist	London
Consultant Neonatologist	Yorkshire & Humber
Consultant Neonatologist	Yorkshire & Humber

Nezafat Maldonado B, *et al. Arch Dis Child Fetal Neonatal Ed* 2024; 109:542–549. doi: 10.1136/archdischild-2023-326152

Supplement 2: Diagnostic codes for CDH

Meets definition if any of the following are present in diagnosis table OR principaldiagnosisatdischarge OR diagnosesday:

10246	Congenital Diaphragmatic Hernia
10694	Morgagni Diaphragmatic Hernia
10905	Recurrent Congenital Diaphragmatic Hernia
11597	Other repair of diaphragmatic hernia
11657	Primary repair of congenital diaphragmatic hernia
11660	Prosthetic repair of congenital diaphragmatic hernia
100044	Congenital Diaphragmatic Hernia
200602	Other specific repair of diaphragmatic hernia
1001921	Repair of diaphragmatic hernia using thoracic approach
1001923	Repair of diaphragmatic hernia using abdominal approach
1001924	Other specific repair of diaphragmatic hernia
1006148	Congenital Diaphragmatic Hernia
1006671	Repair of Congenital Diaphragmatic Hernia
10001925	Unspecified repair of diaphragmatic hernia

Nezafat Maldonado B, *et al. Arch Dis Child Fetal Neonatal Ed* 2024; 109:542–549. doi: 10.1136/archdischild-2023-326152

BMJ Publishing Group Limited (BMJ) disclaims all liability and responsibility arising from any reliance placed on this supplemental material which has been supplied by the author(s)

Supplement 3: Discharge destination, number of neonatal episodes and length of neonatal stay for infants born outside a neonatal surgical unit

First neonatal unit	Discharge destination from neonatal unit	Number of neonatal episodes*		Length of neonatal stay (LOS), days				
			n	Median	Q1	Q3	Minimum LOS	Maximum LOS
Tertiary designated unit	Standalone surgical centre	1	73	2.8	1.5	4.9	0.1	137.3
		2	13	28.3	15.6	34.9	2.1	43.8
		3	1	23.8	23.8	23.8	23.8	23.8
	PICU/CICU	1	50	2.1	1.1	5.4	0.3	28.6
		2	11	34.6	21.3	45.1	7.9	77.1
		3	5	65.1	60.2	123.5	33.7	145.5
		5	1	130.7	130.7	130.7	130.7	130.7
Tertiary non-designated unit	Standalone surgical centre	1	7	0.4	0.3	0.5	0.2	5.0
		2	3	14.1	11.5	28.3	8.9	42.5
		4	2	70.8	43.9	97.6	17.0	124.5
	PICU/CICU	1	2	0.6	0.5	0.6	0.5	0.6
		2	3	25.5	15.3	44.8	5.2	64.2
		3	2	39.9	28.5	51.3	17.1	62.7
		5	1	125	125	125	125	125
Non-tertiary unit	Standalone surgical centre	1	14	0.4	0.3	0.7	0.2	8.2
		2	7	2.2	1.7	4.9	0.3	19.8
		3	1	33.6	33.6	33.6	33.6	33.6
		6	1	66.5	66.5	66.5	66.5	66.5
	PICU/CICU	1	3	0.6	0.5	1.8	0.4	3.1
		2	9	6.9	4	24.6	1.7	54.6
		3	4	36.4	25.1	49.9	7.8	73.7
		4	1	75.1	75.1	75.1	75.1	75.1

**A neonatal episode is defined as a single continuous stay within a single unit.*

Nezafat Maldonado B, *et al. Arch Dis Child Fetal Neonatal Ed* 2024; 109:542–549. doi: 10.1136/archdischild-2023-326152

Supplement 4: Survival to surgical centre for the whole cohort (n=1319)

	Place of Delivery		
	**Tertiary designated unit, N = 337**	**Tertiary non-designated unit, N = 89**	**Non-tertiary unit, N = 233**
**Survival to discharge to surgical centre, n (%)**	261 (77%)	82 (92%)	222 (95%)
**Age transfer to surgical centre, median days (IQR)**	4 (1.8-8)	1 (0.3-1.6)	0.5 (0.3-0.6)

Nezafat Maldonado B, *et al. Arch Dis Child Fetal Neonatal Ed* 2024; 109:542–549. doi: 10.1136/archdischild-2023-326152

BMJ Publishing Group Limited (BMJ) disclaims all liability and responsibility arising from any reliance placed on this supplemental material which has been supplied by the author(s)

Supplement 5: Sub-analysis of discharge outcomes

	**N**	**Discharged to Paediatric ward**	**Discharged to a stand-alone surgical centre/PICU/CICU**	**Discharged to the local hospital**	**Discharged home**	**Length of stay to discharge home**
**Neonatal surgical unit**	660	46 (7%)	67 (10%)	13 (2%)	338 (63%)	27 (16,47)
**Tertiary designated unit**	337	4 (1%)	154 (46%)	1 (3%)	101 (30%)	14 (2-34)
	214	4 (2%)	31 (15%)	1 (0.5%)	101 (47%)	32.3 (20.54-45.4)
**Tertiary non-designated unit**	89	4 (4%)	20 (2%)	0	51 (78%)	19 (12-30)
	80	4 (5%)	11 (14%)	0	51 (64%)	20.3 (16-34.7)
**Non-tertiary unit**	233	5 (2%)	40 (17%)	1 (0.4%)	155 (83%)	19 (11-32)
	216	5 (2%)	23 (10%)	1 (0.5%)	155 (72%)	20.2 (12/6-33/6)

Nezafat Maldonado B, *et al. Arch Dis Child Fetal Neonatal Ed* 2024; 109:542–549. doi: 10.1136/archdischild-2023-326152

Supplement 6: UK Neonatal Collaborative units in England and Wales

Institution	Lead
Airedale General Hospital	Dr Matthew Babirecki
Arrowe Park Hospital	Dr Anand Kamalanathan
Barnet Hospital	Dr Clare Cane
Barnsley District General Hospital	Dr Kavi Aucharaz
Basildon Hospital	Dr Aashish Gupta
Basingstoke & North Hampshire Hospital	Dr Alistair Ewing
Bassetlaw District General Hospital	Dr L M Wong
Bedford Hospital	Dr Anita Mittal
Birmingham City Hospital	Dr Lindsay Halpern
Birmingham Heartlands Hospital	Dr Pinki Surana
Birmingham Women's Hospital	Dr Matt Nash
Bradford Royal Infirmary	Dr Sam Wallis
Broomfield Hospital, Chelmsford	Dr Ahmed Hassan
Calderdale Royal Hospital	Dr Karin Schwarz
Chelsea & Westminster Hospital	Dr Shu-Ling Chuang
Chesterfield & North Derbyshire Royal Hospital	Dr Penelope Young
Colchester General Hospital	Dr Ramona Onita
Conquest Hospital	Dr Graham Whincup
Countess of Chester Hospital	Dr Joanne Dangerfield
Croydon University Hospital	Dr Jocelyn Morris
Cumberland Infirmary	Dr Yee Aung
Darent Valley Hospital	Dr Abdul Hasib
Darlington Memorial Hospital	Dr Mehdi Garbash
Derriford Hospital	Dr Alex Allwood
Diana Princess of Wales Hospital	Dr Pauline Adiotomre
Doncaster Royal Infirmary	Dr Nigel Brooke
Dorset County Hospital	Dr Abby Deketelaere
East Surrey Hospital	Dr Toria Klutse
Epsom General Hospital	Dr Sonia Spathis
Frimley Park Hospital	Sathish Krishnan
Furness General Hospital	Dr Samar Sen
George Eliot Hospital	Dr Jez Jones
Glan Clwyd Hospital	Dr Geedi Farah
Glangwili General Hospital	Dr Prem Pitchaikani
Gloucester Royal Hospital	Dr Jennifer Holman
Good Hope Hospital	Dr Pinki Surana
Great Western Hospital	Dr Stanley Zengeya
Guy's & St Thomas' Hospital	Dr Geraint Lee
Harrogate District Hospital	Dr Sobia Balal
Hereford County Hospital	Dr Cath Seagrave

Nezafat Maldonado B, *et al. Arch Dis Child Fetal Neonatal Ed* 2024; 109:542–549. doi: 10.1136/archdischild-2023-326152

Hillingdon Hospital	Dr Tristan Bate
Hinchingbrooke Hospital	Dr Hilary Dixon
Homerton Hospital	Dr Narendra Aladangady
Hull Royal infirmary	Dr Hassan Gaili
Ipswich Hospital	Dr Matthew James
James Cook University Hospital	Dr M Lal
James Paget Hospital	Dr Oluseun Tayo
Kettering General Hospital	Dr Poornima Pandey
Kings College Hospital	Dr Ravindra Bhat
King's Mill Hospital	Dr Simon Rhodes
Kingston Hospital	Dr Jonathan Filkin
Lancashire Women and Newborn Centre	Dr Savi Sivashankar
Leeds Neonatal Service	Dr Lawrence Miall
Leicester General Hospital	Dr Jonathan Cusack
Leicester Royal Infirmary	Dr Venkatesh Kairamkonda
Leighton Hospital	Dr Michael Grosdenier
Lincoln County Hospital	Dr Ajay Reddy
Lister Hospital	Dr J Kefas
Liverpool Women's Hospital	Dr Alison Bedford Russell
Luton & Dunstable Hospital	Dr Jennifer Birch
Macclesfield District General Hospital	Dr Gail Whitehead
Manor Hospital	Dr Ashok Karupaiah
Medway Maritime Hospital	Dr Ghada Ramadan
Milton Keynes General Hospital	Dr I Misra
Musgrove Park Hospital	Dr Nicola Johnson
New Cross Hospital	Dr Richard Heaver
Newham General Hospital	Dr Mohammad Alam
Nobles Hospital	Dr Prakash Thiagarajan
Norfolk & Norwich University Hospital	Dr Muthukumar
North Devon District Hospital	Dr Tiziana Fragapane
North Manchester General Hospital	Dr Ngozi Edi-Osagie
North Middlesex University Hospital	Dr Cheentan Singh
Northampton General Hospital	Dr Subodh Gupta
Northumbria Specialist Emergency Care Hospital	Jess Reynolds
Northwick Park Hospital	Dr Khadija Ben-Sasi
Nottingham City Hospital	Dr Steven Wardle
Nottingham University Hospital (QMC)	Dr Steven Wardle
Ormskirk District General Hospital	Dr Victoria Nesbitt
Oxford University Hospitals, John Radcliffe Hospital	Dr Eleri Adams
Peterborough City Hospital	Dr Katharine McDevitt
Pilgrim Hospital	Dr Ruchika Gupta
Pinderfields General Hospital (Pontefract General Infirmary)	Dr David Gibson
Poole General Hospital	Prof Minesh Khashu

Prince Charles Hospital	Dr Iyad Al-Muzaffar
Princess of Wales Hospital	Dr Kate Creese
Princess Alexandra Hospital	Dr Chinnappa Reddy
Princess Anne Hospital	Dr Mark Johnson
Princess Royal Hospital	Dr Prashanth Bhat
Princess Royal Hospital (previously Royal Shrewsbury Hospital)	Dr Patricia Cowley
Princess Royal University Hospital	Dr Rashmi Gandhi
Queen Alexandra Hospital	Dr Charlotte Groves
Queen Charlotte's Hospital	Dr Lidia Tyszcuzk
Queen Elizabeth Hospital, Gateshead	Dr Shilpa Ramesh
Queen Elizabeth Hospital, King's Lynn	Dr Glynis Rewitzky
Queen Elizabeth Hospital, Woolwich - see notes	Mrs Julia Croft
Queen Elizabeth the Queen Mother Hospital	Dr Bushra Abdul-Malik
Queen's Hospital, Burton on Trent	Dr Dominic Muogbo
Queen's Hospital, Romford	Dr Ambalika Das
Rosie Maternity Hospital, Addenbrookes	Dr Angela D'Amore
Rotherham District General Hospital	Dr Soma Sengupta
Royal Albert Edward Infirmary	Dr Christos Zipitis
Royal Berkshire Hospital	Dr Peter De Halpert
Royal Bolton Hospital	Dr Archana Mishra
Royal Cornwall Hospital	Dr Chris Warren
Royal Derby Hospital	Dr John McIntyre
Royal Devon & Exeter Hospital	Dr Nagendra Venkata
Royal Hampshire County Hospital	Dr Lucinda Winckworth
Royal Lancaster Infirmary	Dr Joanne Fedee
Royal Oldham Hospital	Dr Anitha Vayalakkad
Royal Preston Hospital	Dr Raju Narasimhan
Royal Stoke University Hospital	Dr Lee Abbott
Royal Surrey County Hospital	Dr Ben Obi
Royal Sussex County Hospital	Dr Prashanth Bhat
Royal United Hospital	Dr Stephen Jones
Royal Victoria Infirmary	Dr Richard Hearn
Russells Hall Hospital	Dr Anjali Petkar
Salisbury District Hospital	Dr Jim Baird
Scarborough General Hospital	Dr Kirsten Mack
Scunthorpe General Hospital	Dr Pauline Adiotomre
Singleton Hospital	Dr Arun Ramachandran
Southend Hospital	Dr Vineet Gupta
Southmead Hospital	Dr Faith Emery
St George's Hospital	Dr Charlotte Huddy
St Helier Hospital	Dr Salim Yasin
St Mary's Hospital, IOW	Dr Akinsola Ogundiya
St Mary's Hospital, London	Dr Lidia Tyszcuzk
St Mary's Hospital, Manchester	Dr Ngozi Edi-Osagie

Nezafat Maldonado B, *et al. Arch Dis Child Fetal Neonatal Ed* 2024; 109:542–549. doi: 10.1136/archdischild-2023-326152

St Michael's Hospital	Dr Pamela Cairns
St Peter's Hospital	Dr Vennila Ponnusamy
St Richard's Hospital	Dr Victoria Sharp
Stepping Hill Hospital	Dr Carrie Heal
Stoke Mandeville Hospital	Dr Sanjay Salgia
Sunderland Royal Hospital	Dr Imran Ahmed
Tameside General Hospital	Dr Jacqeline Birch
The Grange University Hospital	Dr Sunil Reddy
The Jessop Wing, Sheffield	Dr Porus Bastani
The Royal Free Hospital	Dr Marice Theron
The Royal London Hospital - Constance Green	Dr Divyen Shah
Torbay Hospital	Dr Siba Paul
Tunbridge Wells Hospital	Dr Se-Yeon Park
University College Hospital	Dr Giles Kendall
University Hospital Coventry	Dr Puneet Nath
University Hospital Lewisham	Mrs Julia Croft
University Hospital of North Durham	Dr Mehdi Garbash
University Hospital of North Tees	Dr Hari Kumar
University Hospital of Wales	Dr Nitin Goel
Victoria Hospital, Blackpool	Dr Chris Rawlingson
Warrington Hospital	Dr Delyth Webb
Warwick Hospital	Dr Bird
Watford General Hospital	Dr Sankara Narayanan
West Cumberland Hospital	Dr Yee Aung
West Middlesex University Hospital	Dr Elizabeth Eyre
West Suffolk Hospital	Dr Jageer Mohammed
Wexham Park Hospital	Dr Sanjay Jaisal
Whipps Cross University Hospital	Dr Caroline Sullivan
Whiston Hospital	Dr Ros Garr
Whittington Hospital	Dr Wynne Leith
William Harvey Hospital	Dr Vimal Vasu
Worcestershire Royal Hospital	Dr Anna Gregory
Worthing Hospital	Dr Katia Vamvakiti
Wrexham Maelor Hospital	Dr Brendan Harrington
Wythenshawe Hospital	Dr Ngozi Edi-Osagie
Yeovil District Hospital	Dr Megan Eaton
York District Hospital	Dr Sundeep Sandhu
Ysbyty Gwynedd	Dr Michael Cronin

Nezafat Maldonado B, *et al. Arch Dis Child Fetal Neonatal Ed* 2024; 109:542–549. doi: 10.1136/archdischild-2023-326152
